# The Error-Prone Kinetochore-Microtubule Attachments During Meiosis I in Vitrified Oocytes

**DOI:** 10.3389/fcell.2020.00621

**Published:** 2020-07-09

**Authors:** Lei Gao, Yunpeng Hou, Shenming Zeng, Junyou Li, Shien Zhu, Xiangwei Fu

**Affiliations:** ^1^National Engineering Laboratory for Animal Breeding and Key Laboratory of Animal Genetics, Breeding and Reproduction, Ministry of Agriculture, College of Animal Science and Technology, China Agricultural University, Beijing, China; ^2^State Key Laboratory for Agrobiotechnology, College of Biological Sciences, China Agricultural University, Beijing, China; ^3^Animal Resource Science Center, Graduate School of Agricultural and Life Sciences, The University of Tokyo, Ibaraki, Japan

**Keywords:** oocytes vitrification, spindle assembly checkpoint, kinetochore-microtubule attachment, aurora B/C, bivalent stretch

## Abstract

Oocytes vitrification is frequently applied in assisted reproductive technologies. However, chromosomes segregation was error-prone during meiosis maturation of vitrified oocytes. The fidelity of chromosomes segregation depends on the correct kinetochore-microtubule attachments (KT-MTs). In meiosis I, the Aurora B/C would not spatially separate from the attachment sites upon bivalents stretched. Oocytes lack a mechanism for coordinating bivalent stretching and Aurora B/C inhibition in meiosis I. Thus, the KT-MTs are unstable in oocytes. In this study, we firstly found the incorrect KT-MTs were markedly increased in vitrified oocytes. The Aurora B/C activity in vitrified oocytes was significantly increased when the bivalents were stretched. This Aurora B/C activity could not induce a SAC response, as the SAC protein Mad2 was significantly decreased during MI stage in vitrified oocytes. Thus, the KT-MTs in vitrified oocytes were error-prone. This study, for the first time, revealed the mechanism of the incorrect KT-MTs occurred in vitrified oocytes and provided a theoretical basis for further improvement of oocytes vitrification.

## Introduction

The fidelity of chromosomes segregation depends on correct kinetochore-microtubule attachments (KT-MTs) (Tauchman et al., 2015). The KT-MTs could be detached by Aurora B/C during metaphase of meiosis I (MI stage) (Liu et al., 2009; Quartuccio and Schindler, 2015; Yoshida et al., 2015; Wu et al., 2018), which would induce spindle assembly checkpoint (SAC) proteins recruitment (Baker et al., 2004; Zhang et al., 2005; Li et al., 2009; Hached et al., 2011), such as Mad2 (Wassmann et al., 2003; Hached et al., 2011). Mad2 could be recruited onto kinetochores to prevent the activity of anaphase promoting complex/cyclosome (APC/C) (Lischetti and Nilsson, 2015; Vallot et al., 2018), providing an enough time for error correction of KT-MTs. When the KT-MTs were corrected and stabilized, the SAC proteins dissociated from kinetochores, and then the Securin and Cyclin B1 start to be degraded by APC/C, anaphase onsets (London and Biggins, 2014; Yoshida et al., 2015; Marston and Wassmann, 2017; Vallot et al., 2018).

Aurora B/C located in the inner of centromere overlapped with the KT-MT attachment site (Liu et al., 2009). In mitosis, when the kinetochores were correctly attached to microtubules, the tension would drive spatially separation of kinetochores from Aurora B/C, contributing to the stabilization of correct KT-MTs (Lampson and Cheeseman, 2011; Foley and Kapoor, 2013). However, in meiosis I, the Aurora B/C did not spatially separate from the attachment sites during bivalents stretching (Yoshida et al., 2015). Aurora B/C destabilized correct attachments, allowing formation of a considerable amount of incorrect attachments in meiosis I (Kitajima et al., 2011; Yoshida et al., 2015; Touati and Wassmann, 2016). In Meiosis I, the correct KT-MTs were eventually stabilized through PP2A-B56 phosphatase, which was recruited onto kinetochores to counteract Aurora B/C activity (Yoshida et al., 2015). Oocytes lack a mechanism for coordinating bivalent stretching and Aurora B/C inhibition during MI. Thus, the KT-MTs are error-prone in oocytes. Treatment of oocytes with Aurora B/C inhibitor AZD1125 at the middle of the stretching phase (3 h after GVBD), the correct KT-MTs were dramatically increased at the end of the stretching phase (4 h after GVBD) (Yoshida et al., 2015).

When the bivalents are not stretched (0–4 h after GVBD), Mad2 could be recruited onto kinetochores in an Aurora B/C-dependent manner, however, most Mad2 signal disappears when stable KT-MT attachments were formed (6 h after GVBD) (Wassmann et al., 2003; Vallot et al., 2018). low concentration of nocodazole (400 nM) could preserve the spindle sufficiently well, but delay polar body extrusion by causing a SAC response (Wassmann et al., 2003). When kinetochores loss tension, Aurora B/C-dependent error correction takes place, leading to recruit Mad2 onto kinetochores, which delayed cell cycle (Vallot et al., 2018). However, the SAC was not robust enough to provide an enough time for error correction in oocytes, as the incorrect KT-MTs still occurred before bivalents segregation in oocytes (Kitajima et al., 2011).

Since the first pregnancy from human oocytes cryopreservation was succeed in 1986 (Chen, 1986), many advances have been made in oocytes cryopreservation. At present, it is recognized that oocytes vitrification could markedly increase the success rate of oocytes cryopreservation (Cao et al., 2009; Fadini et al., 2009; Smith et al., 2010). However, some studies have demonstrated that oocytes vitrification has a negative effect on spindle (Liu et al., 2017; Pitchayapipatkul et al., 2017), cytoskeleton (Albarracin et al., 2005; Morato et al., 2008; Luciano et al., 2009; Wei et al., 2013), chromosome (Huang et al., 2007; Cheng et al., 2014; Gao et al., 2019) and mitochondria (Moawad et al., 2014; Nohales-Corcoles et al., 2016; Trapphoff et al., 2016; Gao et al., 2017). The abnormal spindle and chromosomes alignment in vitrified oocytes would compromise the fidelity of chromosomes segregation.

In our previous studies, we found that the aneuploidy rate of vitrified MII oocytes was similar with fresh MII oocytes, however, the rate of aneuploidy was significantly increased after meiosis maturation of vitrified GV oocytes (Cheng et al., 2014). Further study showed the amount of abnormal chromosome alignments were significantly increased during meiosis maturation of vitrified GV oocytes (Gao et al., 2019). The previous study have showed that the abnormal spindle in vitrified mouse oocytes was associated with decreased Aurora A protein expression (Doyle et al., 2014). It is still unknown whether vitrification takes an effect on Aurora B/C-dependent error correction of KT-MTs. We analyzed the KT-MTs, bivalent stretch, SAC protein Mad2 and Aurora B/C activity in fresh (F) and vitrified (V) groups. This study would provide a reasonable theoretical basis for further research of meiosis progression in vitrified oocytes.

## Materials and Methods

Unless otherwise stated, all chemicals and media were purchased from Sigma company (United States). All experimental operation of this study were complied strictly in accordance with the ethical principles of animal experimentation approved by Animal Ethics Committee of the China Agricultural University.

### Oocyte Collection

CD1 mice were purchased from Vital River company (Beijing, China) and kept in a room at 20–22°C under every 12 h light cycle and 12 h black cycle. 8–10 weeks old female mice were used for providing GV oocytes.

### Oocytes Vitrification and Warming

Vitrification pretreatment solution consisted of 10% (v/v) dimethylsulfoxide (DMSO), 10% (v/v) ethylene glycol (EG) and 80% PBS. Vitrification solution was made by dissolving 30% Ficoll (w/v), 15% EG (v/v) and 15% DMSO (v/v) in 0.5 M sucrose solution.

The procedure of GV oocytes vitrification was in accordance to the open-pulled straws method (Vajta et al., 1998). Oocytes were treated with pretreatment solution for 30 s and transferred into vitrification solution for 25 s. Then, the open straws with oocytes were immediately put into liquid nitrogen. For thawing of vitrified oocytes, the vitrified oocytes were put into 0.5 M sucrose solution for 5 min and then transferred into M2 medium supplemented with 100 mM dbcAMP. Unless otherwise stated, all manipulations involving oocytes cultured in M2 medium were done on heating plates at 37°C.

### *In vitro* Maturation of Oocytes

GV oocytes were maturated in M16 medium covering with mineral oil, maintaining in an incubator with 5% CO2 and maximum humidity at 37°C. For oocyte treatment with nocodazole: oocytes were transferred in M16 medium containing 400 nM nocodazole at 5 h after GVBD. Oocytes were collected at 6 h after GVBD.

### Microinjection and Live Imaging

Securin-GFP cDNA was a gift from Katja Wassmann. The purified linear Securin-GFP DNA was used as template for RNA production. T3 transcription kit (Thermo, AM1348) was used for Securin-GFP RNA production (Niault et al., 2007). GV oocytes were microinjected with 10 pl of Securin-GFP RNA at 500 ng/μl using an inverted Nikon microscope (Nikon, Japan). Live imaging of oocytes was acquired by a CV1000 system (Yokogawa, Japan) with a 20× objective lens.

### Immunofluorescence

Oocytes (3 and 6 h after GVBD) were transferred in a precooling M2 solution (4°C) for 4 min. Then, oocytes were fixed in a PBS solution containing 5% formaldehyde and 0.3% Triton X-100 at room temperature for 15 min. Oocytes were blocked in 950 μl PBS containing 30 mg BSA and 50 μl 1% Tween20 for 1 h. Then, oocytes were incubated in primary antibody for 1 h at 37°C: human anti-centromere (Immunovision, HCT-0100, 1:200), mouse monoclonal anti-alpha-tubulin with FITC (Sigma, F2168, 1:400), rabbit polyclonal anti-pS55-Hec1 (Genetex, GTX70017, 1:200), and rabbit polyclonal anti-Mad2 (Biolegend, PRB-452C, 1:400). After rinsing three times in PBS, oocytes were transferred into secondary antibodies: anti-human-Cy3 (Jackson ImmunoResearch, AB-2340538, 1:200) and Goat Anti-Rabbit IgG with Fluor 488-labeled (Beyotime, A0423, 1: 400) for 1.5 h at 37°C. Hoechst 33342 (Invitrogen, H21492, 50 mg/mL) was used for Chromosomes stain. Images were acquired using a Nikon A1 confocal laser-scanning microscope (Nikon, Tokyo, Japan) with a 100× objective, scanning *z*-axis at 0.4 mm intervals to span the entire region of the spindle.

### Meiotic Spindle Length and Inter-Kinetochore Distance Measurement

3-D reconstitutions of whole spindles were obtained using NIS-ELEMENTS (Nikon), and the spindle length and inter-kinetochore distance were also detected by NIS-ELEMENTS (Nikon).

### Quantification of pS55-Hec1, Mad2 and Securin-GFP Fluorescence Signal

The fluorescence intensity of pS55-Hec1 Mad2 and Securin-GFP was quantified according to the previous study (Vallot et al., 2018): manually built a 10 × 10 pixel box to put the centromere signal in center and quantified the intensity of centromere, then turn to pS55-Hec1 or Mad2 channel to quantify the intensity; another 10 × 10 pixels box was put adjacent to the first box to quantified the background signal in each channel. As for measuring intensity of each channel signal, the background signal was subtracted. The pS55-Hec1 and Mad2 intensity were normalized to the same kinetochore. For quantitation of Securin-GFP: manually built a 100 × 100 pixel box to put the oocyte Securin-GFP signal in center to quantify the intensity; another 100 × 100 pixels box was put adjacent to the first box to quantified the background signal. As for measuring intensity of Securin-GFP signal, the background signal was subtracted. For each oocyte, values of Securin-GFP were normalized relative to the value of the oocytes (5.5 h after GVBD).

### Western Blot Analysis

200 oocytes from each group were collected at 6 and 9 h after GVBD, respectively. Oocytes with sample buffer were heated at 95°C for 10 min. The sample were loaded onto 10% SDS-PAGE gels for segregating the protein and then transferred onto polyvinylidene fluoride Membranes. The membranes were blocked in 5% (w/ml) fat-free dry milk at room temperature for 1 h. The membranes were incubated with anti-Cyclin B1 (Abcam, ab72, 1:400) and GAPDH antibody (CWBIO, cw0100m, 1:500), respectively, at 4°C overnight, and then washed three times in TBST. The membranes were incubated with Goat anti-mouse IgG (CWBIO, CW0102s, 1:2000) for 1 h at room temperature. Subsequently, immunoreactive signals were detected using an ECL kit (Merck Chemical Co, Germany).

### Statistical Analysis

Graphpad Prism eight software were used for Statistical analysis. Chi-square test was used for analyzing the rates of incorrect KT-MT attachment. Unpaired data was analyzed by two-tailed t tests. The data on Figure 3D was analyzed using Mann-Whitney *U*-test with SPSS (ns represents not significant, ^∗^represents *p* < 0.05, ^∗∗^represents *p* < 0.01, ^∗∗∗^represents *p* < 0.001). Unless otherwise stated, error bars indicated means ± SD.

## Results

### Vitrification Increased the Incorrect KT-MTs During Oocyte Meiosis I

**FIGURE 1:**
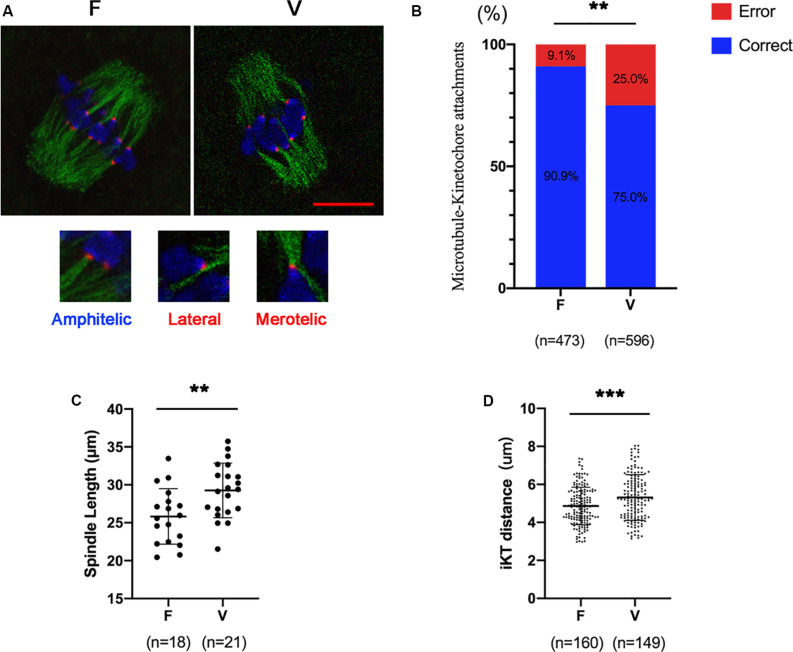
The incorrect KT-MTs were increased in vitrified oocytes. **(A)** Oocytes were fixed at 6h after GVBD to detect kinetochores (red), microtubules (green), and chromosomes (blue). The representative KT-MTs were shown in magnified images, such as correct attachments (end-on amphitelic attachments) and incorrect attachments (one kinetochore attached two microtubules from the opposite spindle polar or a kinetochore attached to the lateral side of microtubule). Scale bar: 10 μm. **(B)** The rate of incorrect KT-MTs was quantified in F and V groups, respectively. The rate of incorrect KT-MTs was significantly increased in vitrified oocytes. F: *n* = 473; V: *n* = 596; *n*: the number of the KT-MT attachments. **(C)** The spindle length in F and V groups were quantified, respectively. F: *n* = 18; V: *n* = 21; *n*: the number of oocytes. **(D)** The distance of kinetochores on each bivalent was measured. F: *n* = 160; V: *n* = 149, *n*: the number of bivalent. The experiments were replicated three times.

Oocytes (6 h after GVBD) were stained to detect kinetochores (red), microtubules (green) and chromosomes (blue; Figure 1A). The correct KT-MT attachments were shown as an end-on amphitelic attachment. The incorrect KT-MTs were shown as merotelic (one kinetochore attached to two microtubules from the opposite spindle polar) and lateral attachments (a kinetochore attached to the lateral side of the microtubule) (Figure 1A). The rate of incorrect KT-MTs was significantly increased in vitrified oocytes compared to fresh oocytes (9.1% vs 25.0%, *p* < 0.01; Figure 1B). The spindle in vitrified oocyte was markedly stretched than fresh oocytes (25.83 ± 3.66 vs 29.26 ± 3.61 μm, *p* < 0.01; Figure 1C). The distance of two kinetochores on each bivalent was significantly increased in vitrified oocytes (4.87 ± 0.99 vs 5.31 ± 1.20 μm, *p* < 0.001), which indicated the bivalents were markedly stretched in vitrified oocytes.

### The APC/C Activity Was Not Inhibited in Vitrified Oocytes

**FIGURE 2:**
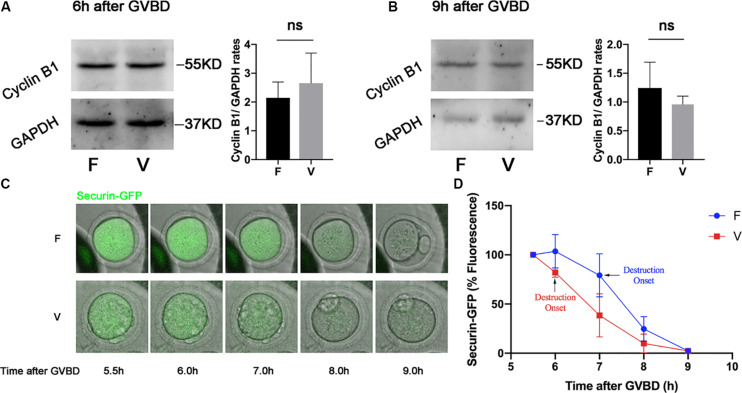
The APC/C activity was not inhibited by vitrification. The protein level of Cyclin B1 were examined by western blot at 6 h **(A)** and 9 h **(B)** after GVBD. The experiments were replicated three times. The Cyclin B1 was quantified in both F and V group. **(C)**. Securin-GFP RNA was injected into GV oocytes, and the fluorescence intensity was monitored during meiosis maturation of oocytes. The image of Securin-GFP signal was acquired by time-lapse fluorescence imaging. **(D)** The dynamic of Securin-GFP fluorescence was measured from 5.5 to 9 h after GVBD, F: *n* = 14, V: *n* = 10; *n*: the number of oocytes. The experiments were replicated three times.

Cyclin B1 and Securin were in a high level at 6 h after GVBD, and were degraded to the lowest level at 9 h after GVBD, which was induced by the activation of APC/C (Nabti et al., 2014). The Cyclin B1 in oocytes was measured by western blot at 6 h after GVBD (Figure 2A), the Cyclin B1 level in vitrification group was similar with fresh group (2.15 ± 0.55 vs 2.66 ± 1.04, *p* > 0.05; Figure 2A). We also detected the Cyclin B1 level in oocytes at 9h after GVBD (Figure 2B). The Cyclin B1 level in vitrification group was comparable to that in fresh group at 9 h after GVBD (1.24 ± 0.45 vs 0.96 ± 0.14, *p* > 0.05). The Securin-GFP was also decreased since 6 h after GVBD in both fresh and vitrified oocytes (Figure 2C), suggesting the APC/C activity was not inhibited in vitrified oocytes. The highest value of Securin-GFP was at 6h after GVBD in fresh oocytes, and was at 5.5 h after GVBD in vitrified oocytes. The Securin-GFP destruction onset time was at 7 h after GVBD in fresh oocytes, and was at 6 h after GVBD in vitrified oocytes (Figure 2D).

### Vitrified Oocytes Failed to Maintain the SAC Activity During MI

**FIGURE 3:**
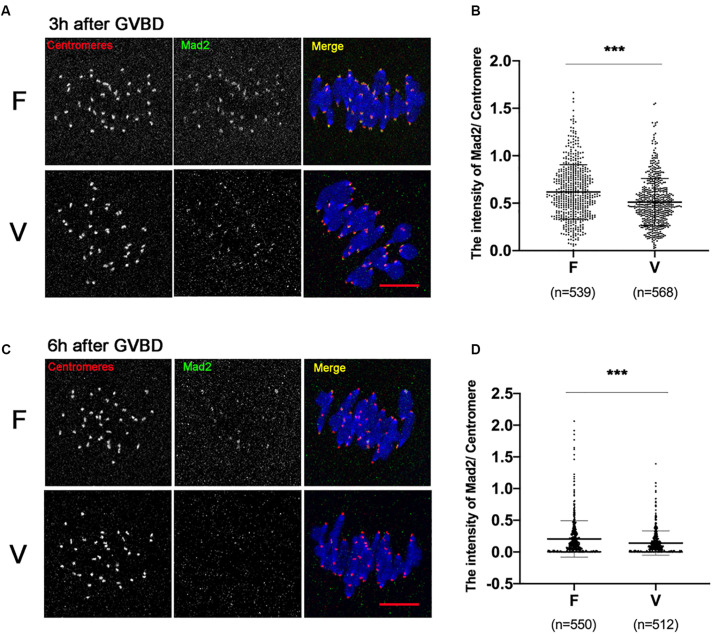
The SAC was significantly decreased in vitrified oocytes at 6 h after GVBD. **(A)** 19 fresh oocytes and 19 vitrified oocytes were fixed at 3 h after GVBD to detect centromeres (Red), Mad2 (Green) and chromosomes (Blue). Scale bar: 10 μm. **(B)** The intensity of Mad2/Centromere were quantified at 3h after GVBD. F, *n* = 539; V, *n* = 568, *n*: the number of the Centromere. **(C)** 18 fresh oocytes and 16 vitrified oocytes were fixed at 6 h after GVBD to detect centromeres (Red), Mad2 (Green) and chromosomes (Blue). Scale bar represents 10 μm. **(D)** The intensity of Mad2/Centromere was quantified at 6 h after GVBD. F: *n* = 550; V: *n* = 512, *n*: the number of the Centromere. The experiments were replicated three times.

SAC protein Mad2 could be recruited onto kinetochores at 0–4 h after GVBD, and most of Mad2 signal disappears when stable KT-MT attachments were formed (6 h after GVBD). We detected the Mad2 level on kinetochores at 3 h after GVBD (Figure 3A). Comparing to fresh oocytes, the Mad2 signal was markedly decreased in vitrified oocytes at 3h after GVBD (0.62 ± 0.29 vs 0.51 ± 0.25, *p* < 0.001; Figure 3B). The Mad2 level on kinetochores was also detected at 6 h after GVBD (Figure 3C). The Mad2 signal was significantly decreased in vitrified oocytes (0.21 ± 0.29 vs 0.14 ± 0.19, *p* < 0.001; Figure 3D). These results indicated that SAC was not activated even an increase of incorrect KT-MTs in vitrified oocytes.

### The SAC Could Be Recruited Onto Kinetochores After Treatment With Low Concentration of Nocodazole

**FIGURE 4:**
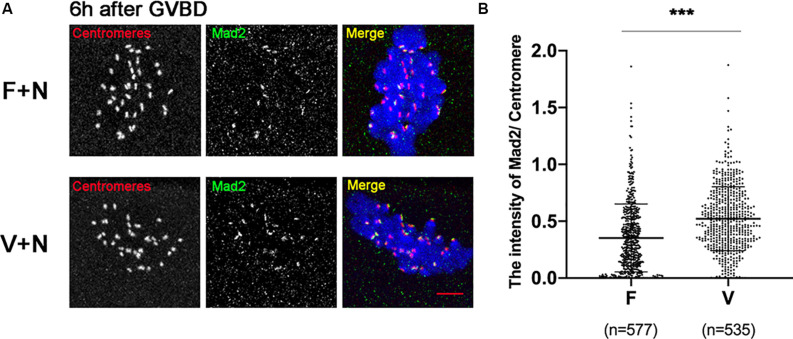
Mad2 was recruited onto kinetochores after treatment with nocodazole. **(A)** 16 fresh oocytes and 16 vitrified oocytes were treated with nocodazole (400 nM) at 5 h after GVBD. Then, ooctyes were fixed at 6 h after GVBD to detect centromeres (Red), Mad2 (Green), and chromosomes (Blue). Scale bar: 5 μm. **(B)** The intensityof Mad2/Centromere was quantified in F+N (fresh oocytes treated with nocodazole) and V+N (vitrified oocytes treated with nocodazole) group. F+V: *n* = 577; V+N: *n* = 535, *n*: the number of the Centromere. The experiments were replicated three times.

low concentration of nocodazole (400 nM) could preserve the spindle sufficiently well and induce a SAC response due to destabilize the KT-MTs (Wassmann et al., 2003). Treatment with 400nM nocodazole, the Mad2 was recruited onto kinetochores in oocytes at 6 h after GVBD (Figure 4A). The Mad2 was significantly increased in V+N (Vitrified oocytes treated with nocodazole) group compared to F+N (fresh oocytes treated with nocodazole) group (0.35 ± 0.30 vs 0.52 ± 0.28, *p* < 0.001; Figure 4B), which indicated the SAC could be activated by destabilizing KT-MTs in vitrified oocytes.

### The Aurora B/C Activity Did Not Reach the Threshold of Recruiting SAC in Vitrified Oocytes

pS55-Hec1 is an Aurora B/C target on the kinetochores. pS55-Hec1 would be decreased upon inhibition of Aurora B/C (Yoshida et al., 2015; Vallot et al., 2018). The signal intensity of pS55-Hec1 was significantly increased in vitrified oocytes at 6 h after GVBD (0.28 ± 0.20 vs 0.41 ± 0.21, *p* < 0.001; Figures 4A,B), which indicated the activity of Aurora B/C was increased in vitrified oocytes. However, this Aurora B/C could not induce a SAC response in vitrified oocytes. Treatment of oocytes with 400 nM nocodazole to destabilize the KT-MTs, the pS55-Hec1 signal in V+N group was significantly increased compared to V group (0.41 ± 0.21 vs 0.58 ± 0.22, *p* < 0.001; Figures 4A,B). These results indicated that the Aurora B/C activity in vitrified oocytes was significantly increased, however, this Aurora B/C activity could not induce a SAC response.

## Discussion

The fidelity of chromosome segregation depends on the correct KT-MTs (Tauchman et al., 2015). The chromosome segregation errors were markedly increased in vitrified oocytes (Cheng et al., 2014; Gao et al., 2019). Consistently, the incorrect KT-MTs were significantly increased in vitrified oocytes (Figures 1A,B). Most of incorrect KT-MTs could be corrected during MI stage (Kitajima et al., 2011), which would induce a SAC response to delay cell cycle. However, the SAC was not robust enough in oocytes (Kolano et al., 2012; Baumann et al., 2017), as the KT-MTs errors still occurred before bivalents segregation (Kitajima et al., 2011). We also found some incorrect attachments in fresh oocytes at 6h after GVBD (Figures 1A,B).

The incorrect KT-MTs would induce Aurora B/C-depended error correction, which resulted in a SAC response (Welburn et al., 2010; Yoshida et al., 2015; Vallot et al., 2018). When SAC was activated, the degradations of Cyclin B1 and Securin would be inhibited and the first polar body cannot be extruded (Clute and Pines, 1999; Zur and Brandeis, 2001; Herbert et al., 2003; Terret et al., 2003). However, the polar body extrusion was not delayed in vitrified oocytes (Gao et al., 2019). Consistently, the degradation of Cyclin B1 and Securin-GFP was not prevented in vitrified oocytes (Figure 2), suggesting that SAC was not strong enough to inhibit the activation of APC/C in vitrified oocytes. In addition, securin-GFP destruction onset was accelerated in vitrified oocytes, which indicated the SAC may be not robust in vitrified oocytes (Figure 2D).

The SAC was recruited on kinetochores at prometaphase of meiosis I (0-4h after GVBD) and most of SAC signals were disappeared when correct KT-MTs formed on stretched bivalents at later of MI stage (6 h after GVBD) (Wassmann et al., 2003; Hached et al., 2011; Vallot et al., 2018). Compared to fresh oocytes, the SAC protein Mad2 was markedly decreased in vitrified oocytes at MI stage (Figure 3). Consistently, the previous study also found the Mad2 mRNA content was significantly decreased at MI stage in vitrified oocytes (Wu et al., 2019). Fewer SAC signals on kinetochores were not sufficient to prevent the activity of APC/C, which failed to provide an enough time for error correction of KT-MTs in vitrified oocytes.

The SAC was recruited onto kinetochores through an Aurora B/C-dependent destabilization of KT-MTs (Kitajima et al., 2011; Yoshida et al., 2015; Touati and Wassmann, 2016). Treatment of oocytes with low concentration of nocodazole (400nM), which caused a SAC response due to destabilize the KT-MTs (Wassmann et al., 2003). The Mad2 signal was significantly increased in vitrified oocytes after treatment with nocodazole (Figure 4). This result indicated that the decline of SAC recruitment was due to the difficulty to destabilize the incorrect KT-MTs in vitrified oocytes.

**FIGURE 5:**
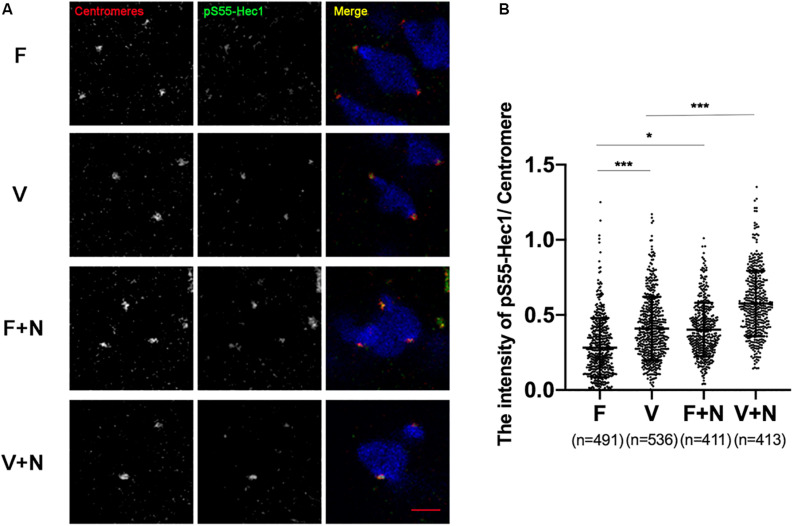
The activity of Aurora B/C in vitrified oocytes did not reach the threshold of recruiting SAC. **(A)** We treated oocytes with 400 nM nocodazole at 5 h after GVBD and fixed oocytes at 6 h after GVBD to detect chromosomes (Blue), centromeres (Red), and pS55-Hec1 (Green). 22 oocytes in F group, 19 oocytes in V group, 19 oocytes in F+N group and 18 oocytes in V+N group were detected. Scale bar represents 2 μm. **(B)** The intensity of pS55-Hec1/centromere was quantified in F, V, F+N, and V+N group, respectively. F: *n* = 491; V: *n* = 536; F+N: n = 411; V+N: *n* = 413, *n*: the number of the Centromere. The experiments were replicated three times.

Phosphorylated Hec1 at S55 (pS55-Hec1) is an Aurora B/C target on the kinetochores for destabilizing KT-MTs (Cheeseman et al., 2006; DeLuca et al., 2006). Inhibition of Aurora B/C decreases the level of pS55-Hec1 (Vallot et al., 2018). During 2–4 h after GVBD, the bivalents stretch and establish a fully bioriented state in oocytes. The level of pS55-Hec1 at the kinetochores is significantly increased from 2 to 4 h after GVBD, however, the amount of pS55-Hec1 on stretched bivalents at 6 h after GVBD was significantly lower than that observed at 4 h after GVBD (Yoshida et al., 2015). During the stabilization phase (4–6 h after GVBD), pS55-Hec1 is dephosphorylated via the CDK1-BubR1-PP2A pathway, stabilizing the KT-MT attachments (Yoshida et al., 2015). Consistently, the Mad2 was recruited onto kinetochores during 2–4 h after GVBD and disappeared from kinetochores at 6 h after GVBD (Wassmann et al., 2003). Since the KT-MTs were more stabilized at 6 h after GVBD than that at 2–4 h after GVBD and the Aurora B/C activity was significantly lower at 6 h after GVBD than that at 4 h after GVBD (Yoshida et al., 2015), the increased Aurora B/C activity in vitrified oocytes could not destabilize KT-MTs and induce an increase of Mad2 (Figures 3D, 5B).

In order to prove whether the above hypothesis was true, we treated vitrified oocyte with low concentration of nocodazole (400 nM) at 5 h after GVBD and detected pS55-Hec1 and Mad2 at 6 h after GVBD. High concentration of nocodazole would completely remove oocytes spindle. However, low concentration of nocodazole (400 nM) could preserve the spindle sufficiently well and allow polar body extrusion after longer exposure, which caused a SAC response due to destabilize the KT-MTs or loss of tension (Wassmann et al., 2003). When KT-MTs miss tension, Aurora B/C-dependent error correction took place (Vallot et al., 2018). The Aurora B/C activity was significantly increased and the Mad2 was significantly recruited onto kinetochores in vitrified oocytes after treatment with low concentration of nocodazole (400 nM, Figures 4, 5). This result indicated that Aurora B/C activity could not induce a SAC response in vitrified oocytes at 6 h after GVBD.

## Conclusion

**FIGURE 6:**
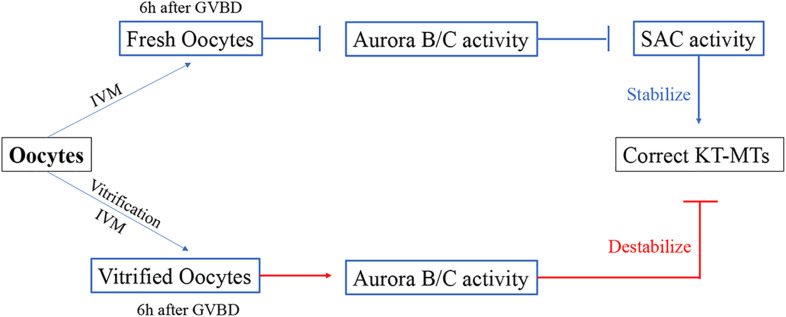
Model for the formation of incorrect KT-MTs in vitrified oocytes. The Aurora B/C activity were significantly increased when bivalents were markedly stretched at 6h after GVBD. This Aurora B/C activity could not induce a SAC response, as the SAC protein Mad2 was significantly decreased during MI stage in vitrified oocytes. Thus, the KT-MTs were error-prone in vitrified oocytes.

Our results showed that the incorrect KT-MTs were markedly increased in vitrified oocytes. This frequent error of KT-MTs may be induced by an increase of Aurora B/C activity. However, this Aurora B/C activity could not induce a SAC response, as the SAC protein Mad2 was significantly decreased during MI stage in vitrified oocytes. Thus, the KT-MTs were error-prone in vitrified oocytes (Figure 6).

## Data Availability Statement

All datasets generated for this study are included in the article/supplementary material.

## Author Contributions

XF and LG designed the research, analyzed the data and wrote the manuscript. LG performed the *in vitro* maturation, oocytes vitrification, Immunofluorescence, Western blot, microinjection and live imaging. YH, SZ, JL, and SZ interpreted the results. All authors contributed to the article and approved the submitted version.

## Conflict of Interest

The authors declare that the research was conducted in the absence of any commercial or financial relationships that could be construed as a potential conflict of interest.
